# Spectrophotometric Assay for the Detection of 2,5-Diformylfuran and Its Validation through Laccase-Mediated Oxidation of 5-Hydroxymethylfurfural

**DOI:** 10.3390/ijms242316861

**Published:** 2023-11-28

**Authors:** Nicoletta Cascelli, Vicente Gotor-Fernández, Iván Lavandera, Giovanni Sannia, Vincenzo Lettera

**Affiliations:** 1 Biopox srl, Viale Maria Bakunin 12, 80125 Napoli, Italy; cascellinicoletta@uniovi.es (N.C.); sannia@biopox.com (G.S.); 2 Organic and Inorganic Chemistry Department, University of Oviedo, Avenida Julián Clavería 8, 33006 Oviedo, Spain; vicgotfer@uniovi.es (V.G.-F.); lavanderaivan@uniovi.es (I.L.); 3 Dipartimento di Chimica e Tecnologie Chimiche, Università della Calabria, Via P. Bucci Cubo 12/D, 87036 Cosenza, Italy

**Keywords:** colorimetric assay, 2,5-diformylfuran, colorimetric screening, 5-hydroxymethylfurfural, laccase–mediator system

## Abstract

Modern biocatalysis requires fast, sensitive, and efficient high-throughput screening methods to screen enzyme libraries in order to seek out novel biocatalysts or enhanced variants for the production of chemicals. For instance, the synthesis of bio-based furan compounds like 2,5-diformylfuran (DFF) from 5-hydroxymethylfurfural (HMF) via aerobic oxidation is a crucial process in industrial chemistry. Laccases, known for their mild operating conditions, independence from cofactors, and versatility with various substrates, thanks to the use of chemical mediators, are appealing candidates for catalyzing HMF oxidation. Herein, Schiff-based polymers based on the coupling of DFF and 1,4-phenylenediamine (PPD) have been used in the set-up of a novel colorimetric assay for detecting the presence of DFF in different reaction mixtures. This method may be employed for the fast screening of enzymes (Z’ values ranging from 0.68 to 0.72). The sensitivity of the method has been proved, and detection (8.4 μM) and quantification (25.5 μM) limits have been calculated. Notably, the assay displayed selectivity for DFF and enabled the measurement of kinetics in DFF production from HMF using three distinct laccase–mediator systems.

## 1. Introduction

**Figure 1:**
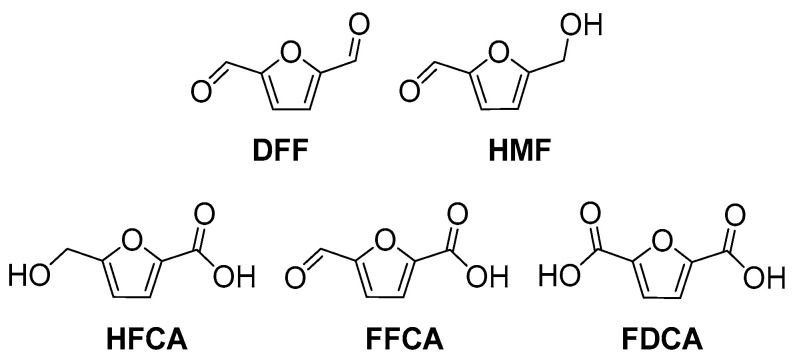
Structures of the tested compounds: DFF, 2,5-diformylfuran; HMF, 5-hydroxymethylfurfural; HFCA, 5-hydroxymethyl-2-furancarboxylic acid; FFCA, 5-formyl-2-furancarboxylic acid; FDCA, 2,5-furandicarboxylic acid.

Cellulosic sugars are currently the subject of extensive studies for their conversion into furan derivatives [1,2,3,4], which are heterocyclic compounds with great possibilities as alternatives to compounds derived from fossil resources in the energy and chemical industry. Amongst all of these bio-based compounds, 2,5-diformylfuran (DFF, Figure 1) has received considerable attention due to the wide range of potential applications in the synthetic industry as a renewable and versatile building block [5]. Containing a furan ring and two aldehyde groups, DFF is a valuable substrate for polymer synthesis to obtain materials such as plastics, coatings, adhesives, and resins [6,7,8], as well as pharmaceuticals [9,10]. DFF can be condensed with other molecules to make, for instance, poly-imines [7,11,12], polyesters, polyamides and polyurethanes [13], and polymers that display unique properties in performances that compete with those from petroleum-based materials [14]. For this reason, DFF is considered as a bridge connecting renewable biomass and the petroleum industry. This compound offers sustainable alternatives to chemicals employed in the pharmaceuticals, fuel, and polymers sectors, and it is currently commonly derived from fossil source manufacturing processes [15]. A noteworthy application of DFF was revealed by Dhers et al., who demonstrated the synthesis of a polyimine vitrimer used in dynamic film design, boasting an entirely renewable carbon content of 100% [7]. Several other valuable DFF derivatives have found industrial applications, for instance, furan-2,5-dicarbonyl chloride (FDCC) [16], 2,5-bis(aminomethyl)furan (BAMF) [17], 2,5-bis(hydroxymethyl)-tetrahydrofuran [18], 2,5-dicyanofuran [19], and 2,5-bis(aminomethyl)furan [20]. Moreover, DFF is also investigated as a potential platform for the production of high-value specialty chemicals that can be used as solvents or further processed to produce biofuels such as biodiesel and jet fuel [21,22,23].

DFF can be efficiently synthesized under aerobic conditions from 5-hydroxymethylfurfural (HMF, Figure 1), one of the most versatile furan-based compounds, derived from biomass-based feedstock [1,24,25]. The selective conversion of HMF into DFF, via homogeneous or heterogeneous catalysis, has been widely reported [26,27,28]. DFF chemical syntheses via traditional oxidative catalytic processes involve base-metal-containing heterogeneous catalysts, mainly from ruthenium [29], vanadium [30], manganese [31], or copper [32], among others, either in aqueous or organic media [33]. Nevertheless, these processes require, in some cases, several reaction steps, expensive catalysts and hazardous organic solvents, and a careful control of harsh reaction conditions, such as high temperatures and pressures, to avoid the formation of overoxidized co-products, for instance, HFCA, FFCA, and FDCA (Figure 1) [34,35].

In addition to these traditional catalytic processes, more environmentally friendly transformations have been investigated towards DFF synthesis. More recently, the conversion of renewable and cheap carbohydrates (i.e., fructose) has been proposed as a sustainable approach to produce this compound [36]. Additionally, new strategies involving biocatalysis [37,38,39,40,41], photocatalysis [42,43,44,45], and electrocatalysis [46,47] starting from HMF have been embraced. Among all, enzymatic oxidations are extensively reported, showing, in some cases, an excellent selectivity for DFF formation over other possible oxidized derivatives. Several enzymes have efficiently performed HMF oxidation using oxygen or other oxidants as electron acceptors. Glucose oxidases [48], copper radical oxidases [49], aryl-alcohol oxidases [41,50], and HMF oxidases [51] are the most appealing so far, while galactose oxidase (GOx) is the most reported [52].

Among oxidative enzymes, laccases have also been utilized in the oxidation of HMF. Laccases are copper-containing oxidoreductases that exist naturally in many organisms, which catalyze the oxidation of a wide range of phenolic compounds, aromatic amines, and other substrates by reducing molecular oxygen to water. They have found applications in biosensors, pulp and paper manufacturing, organic synthesis, textiles, cosmetics, and both synthetic and degradative processes, highlighting their significance across multiple industrial sectors [53,54,55].

Laccases often exhibit low activity towards non-phenolic substrates such as alcohol derivatives and therefore face challenges in directly oxidizing certain derivatives. For this reason, these enzymes must be combined with small molecules, forming a laccase–mediator system (LMS). The mediator oxidized by the laccase acts as an intermediate species between the enzyme and the target substrate, mediating the electron transfer towards the substrate that is otherwise difficult to oxidize. Finally, the laccase is activated again by the oxygen from air [56,57,58,59,60].

Despite that, the selective synthesis of DFF has proven to be quite challenging; none of the proposed enzymatic systems are practical solutions for high-scale DFF production, among other factors, due to the solubility, selectivity, and stability issues of the selected biocatalysts. There are still some challenges ahead in the development of biotransformations, such as productivity and scalability. In order to fine-tune enzymes or boost their catalytic activity and operational stability, protein engineering has been proposed as an enabling technology. In general, biocatalyst engineering strategies include directed evolution and site-directed mutagenesis, two powerful tools that can be used, alternatively or simultaneously, to optimize and diversify enzyme functions for a wide range of applications [61,62,63]. These methodologies have been successfully pursued to improve the properties of many different proteins [64]. With this scenario, the implementation of rapid and selective high-throughput screenings is a need to test up to millions of enzyme variants towards a specific transformation [65,66].

As already mentioned, the search for new enzymes that are able to oxidize HMF into DFF may open new unexplored biocatalytic routes. The main obstacle in approaching these strategies is related to the lack of a fast and sensitive screening method for specific DFF identification. To date, the most common related high-throughput assays deal with the detection of co-products derived from the oxidation of HMF. In this context, one of the most representative contributions is the one reported by Turner and co-workers [67], who generated a library of GOx variants via the site-directed mutagenesis of selected residues in the active site of the enzyme, finding a suitable catalyst for HMF oxidation. This library was screened towards this substrate by measuring the amount of the co-product H_2_O_2_ through a coupled peroxidase-based colorimetric reaction via the co-oxidation of 4-chloronaphthol or ABTS [68]. This assay allowed for the best performing hits to be selected without any selective detection of DFF, which was only measured using analytical methods (GC and HPLC) for the selected variants. This approach, however, cannot be employed when working with enzymes that do not generate hydrogen peroxide as a co-product.

As far as we know, a versatile screening methodology for the selective direct DFF detection has not yet been reported. In this contribution, we developed an assay to be used as a fast method to reveal DFF in a solution, with a special focus on the chemical selectivity. The design of this rapid assay for DFF revelation is proposed to be consisting of the polymeric condensation of this dialdehyde with an aromatic diamine, namely 1,4-phenylenediamine (*p*-phenylenediamine, PPD) [69]. The assay aims to provide a linear and sensitive response to the analyte, avoiding interferences with the starting material and other possible co-products. Moreover, the effectiveness in the synthesis of DFF of three distinct LMSs was tested through the assay.

## 2. Results and Discussion

### 2.1. Rationale and Set-Up of the Assay Methodology towards DFF

The selective oxidation of HMF into DFF is gaining increasing importance since it is considered an ideal platform for producing value-added compounds. Unlike traditional chemical oxidations, the enzymatic conversion of HMF is preferred, assuring high selectivity in DFF synthesis as well as a more sustainable process. However, enzymes actively performing this transformation are scarce. In this regard, directed evolution studies may impart a pivotal contribution by enhancing the promiscuous activities of natural enzymes as well as by enabling the screening of numerous new biocatalysts. The design of such complex approaches can be limited mainly by the absence of sensitive and easy high-throughput screening technologies. Thus, a fast and simple analytical method for the selective detection of DFF in mixtures is needed. Few detection methods, including colorimetric and fluorometric ones, have already been reported for several furan-based compounds [70,71], including HMF [72,73]. Conversely, DFF has never been considered as the main target for the design of such assays, despite the increasing interest in its selective synthesis from HMF.

Polymeric Schiff bases, formed mainly through the reaction of diamines with dialdehydes such as DFF, have already been disclosed [74,75,76]. All of these studies have been proposed as proof-of-concept for the preparation of new bio-based polymeric materials. Whereas the characterization of DFF-based poly-imines has been presented as promising polymers with relevant conducting properties [69], or forming furan-based porous organic frameworks [11], they have not been effectively exploited for their colorimetric properties in the design of, e.g., a selective assay for DFF detection.

**Scheme 1:**
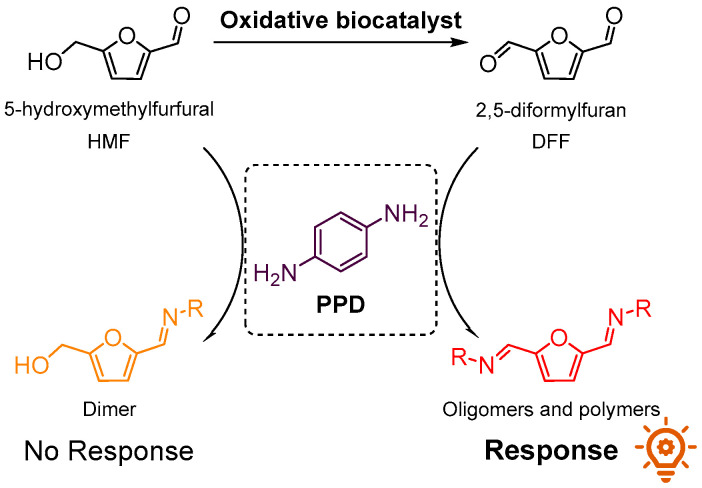
Representation of the concept of the designed screening assay described in this contribution. HMF oxidation leads to DFF accumulation. Then, PPD incubation can selectively reveal DFF.

The herein developed method is based on the selective reaction of DFF with the aromatic diamine PPD, exploiting its reactivity towards the target compound. The assay is designed to reveal DFF as an oxidation product from HMF. Nevertheless, the aldehyde moiety of HMF could also react with PPD to form the corresponding imine, which could undergo dimerization but no further polymerization due to the presence of the hydroxyl functionality, although it might contribute to the formation of color. In order to set-up a selective colorimetric screening for DFF, the conjugated structures obtained via PPD incubation with HMF must not interfere with the absorbance of DFF in the UV/Vis region (Scheme 1).

**Figure 2:**
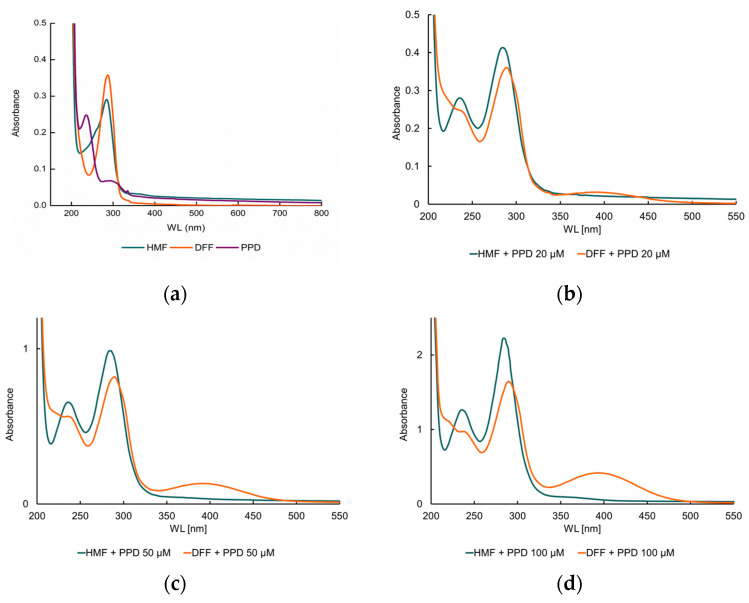
UV/Vis spectra of (**a**) HMF, DFF, and PPD (20 μM each); HMF (substrate) or DFF (product) at (**b**) 20, (**c**) 50, and (**d**) 100 μM incubated with PPD (same concentration) in KPi buffer 100 mM, pH 7.5.

Since this assay is intended to be applied in biocatalytic oxidative transformations, the incubation occurred in an aqueous medium at a slightly basic pH (KPi buffer 100 mM, pH 7.5), thus favoring the formation of the Schiff base. As a first step, the spectrophotometric UV/Vis characterization of HMF, DFF, and PPD alone (equimolar mixtures, 20 μM) confirmed that the compounds singularly did not significantly absorb in the visible region (above 380 nm, Figure 2a). Thus, HMF and DFF were separately incubated in the presence of PPD, using up to 100 μM of each compound. A positive response was only detected for DFF at a wavelength of 350–500 nm (Figure 2b–d).

**Figure 3:**
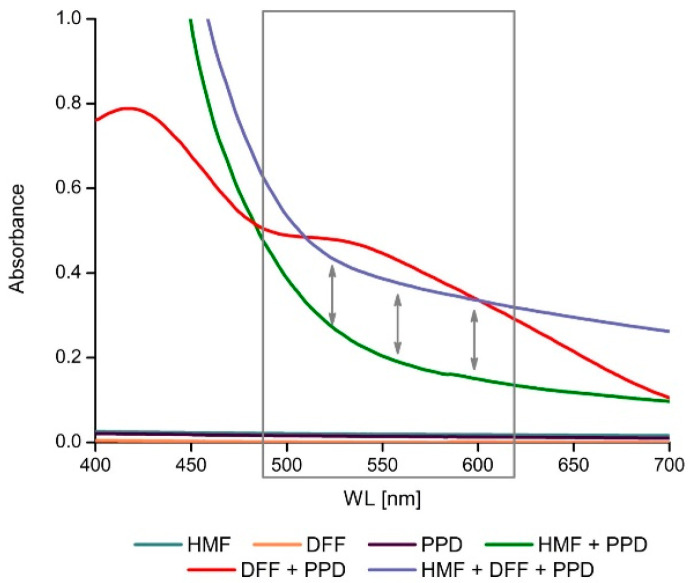
UV-Vis spectra of HMF, DFF, PPD, and their mixtures. The difference in absorbance when DFF was added to the mixture of HMF and PPD is highlighted with grey arrows inside the rectangle.

Then, a simulated reaction progress with 2% HMF conversion into DFF was mimicked by mixing HMF (4.9 mM) and DFF (100 μM, Figure 3). The single compounds were incubated with PPD (5 mM), as well, for comparison. Initially, assays were conducted in 1 mL of the total volume at room temperature. The profile peak of DFF reacting with PPD (red line, Figure 3) reflected the previous evidence of the shift in the absorbance detected at 450–500 nm instead of the lower absorbance values when reacting HMF with PPD (green line, Figure 3), which was set as the background noise of the assay under the explored conditions. The same trend at wavelengths of 500–600 nm was observed when HMF reacted with PPD in the presence of such little amount of DFF (purple line, Figure 3), with an increase in the overall absorbance values due to the contribution of the polymer formed between DFF and PPD.

**Figure A1:**
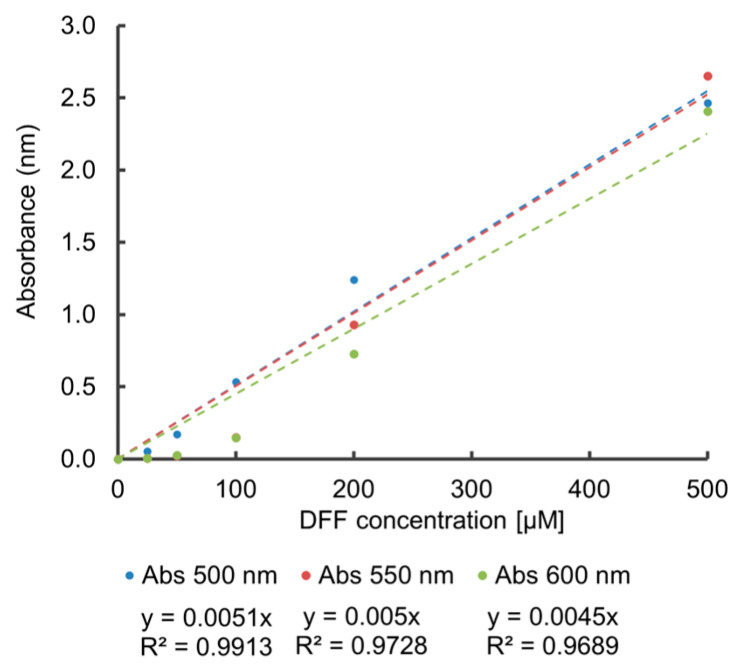
Absorbance measurements at 500, 550, and 600 nm at increasing DFF concentrations in the solution. The experiments were performed in a 96-well multi-plate.

A point-by-point evaluation of the difference in increasing absorbance, attributed to the presence of DFF even in the presence of a high excess of HMF (indicated by the purple line), revealed comparability. Three values within this range (500, 550, and 600 nm) were selected, and the linearity of the response in the presence of increasing DFF concentrations was assessed. After confirming the suitability of all three wavelengths for detecting DFF (Appendix A, Figure A1), 500 nm was chosen, which was also due to the lower R^2^ value, to validate the method and investigate the sensitivity of the assay.

**Figure 4:**
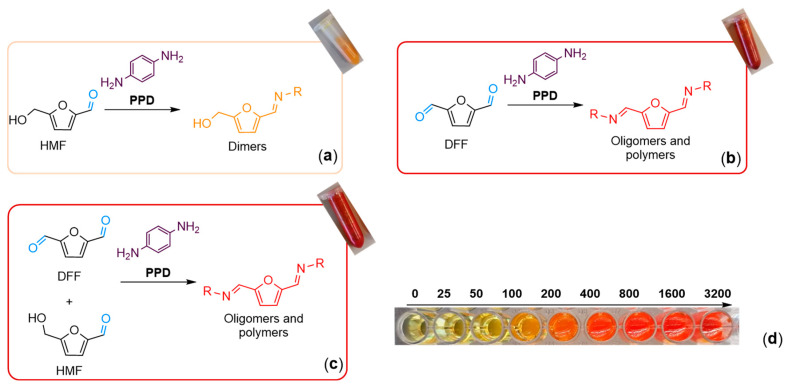
Different colors of the precipitates formed when PPD (1 equivalent) was incubated with HMF—panel (**a**); DFF—panel (**b**); or in an HMF:DFF equimolar mixture—panel (**c**). A shift in color was observed at increasing DFF concentrations (units in μM in the in vitro system—panel (**d**)).

Likely, the formation of Schiff polymers derived from the reaction of DFF in the presence of PPD, even in small amounts, accounted for the major color that was formed (Figure 4). While the development of an orange color was revealed with only HMF (panel a), the formation of a red precipitate was clear when PPD was incubated with DFF (panel b), or in a mixture of HMF and DFF (panel c). Moreover, the color intensity in the assay was obviously related to the DFF amount, as higher concentrations led to intensely red-colored solutions (panel d).

### 2.2. Linearity and Sensitivity of the Assay

**Figure 5:**
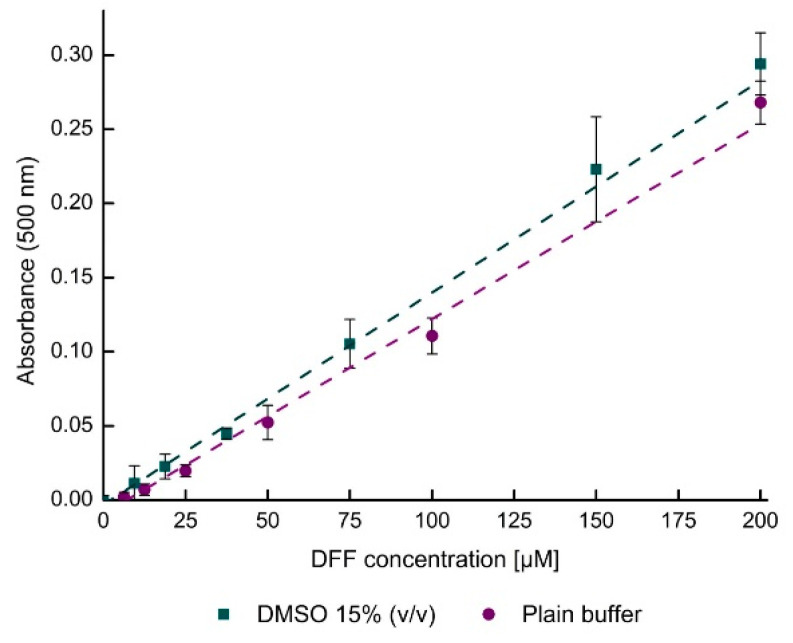
Linearity of the colorimetric assay at different concentrations of DFF (0–200 µM) in plain buffer (KPi 100 mM, pH 7.5, purple line) or with DMSO (15% *v*/*v*, green line). The data shown are representative of experiments performed in triplicate.

The linearity of the assay was assessed by measuring the variation of the absorbance at increasing analyte concentrations (Figure 5). Nevertheless, DFF synthesis from HMF has proven to be efficient in the presence of organic (co)solvents, mainly due to the solubility limits of furans in aqueous buffer systems [77]. Moreover, thinking about the scalability of biocatalytic systems, there will be a need for cosolvents for HMF and DFF solubilization at higher concentrations. Amongst all of the reported solvents commonly investigated for the dehydration of carbohydrates and HMF oxidation, dimethylsulfoxide (DMSO) was chosen for further investigation [78,79,80]. However, DMSO interfered with DFF detection, slowing down the response; thus, the DMSO concentration was fixed to 15% *v*/*v*.

DMSO has a beneficial effect on organic substrate solubilities, providing a one-phase system, and its efficiency in enzymatic reactions has been previously proven with many enzyme classes (ADHs, ATAs, etc.) [81,82,83]. Also, the three laccases were previously efficiently tested at high DMSO concentrations, revealing a good stability [84,85]. Gladly, when performing the assay under these reaction conditions, no relevant interference was observed. Thus, the linearity of the assay was proven in both plain buffer (KPi 100 mM, pH 7.5) and DMSO buffer systems up to 200 μM of DFF (Figure 5). In the latter, the slope of the curve obtained from the regression analysis was even slightly improved, suggesting a DFF solubility limit in a plain buffer and a more homogeneous system in the presence of DMSO.

**Figure A2:**
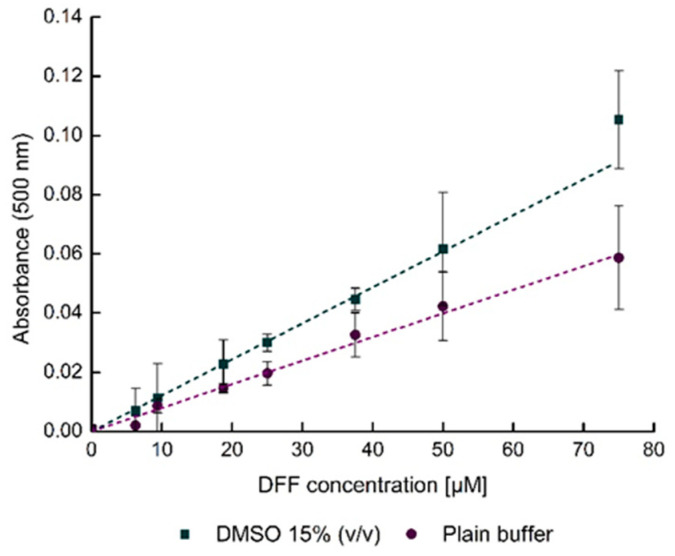
Linearity of the system and its sensitivity (LOD and LOQ) were determined at low DFF concentrations. The data shown are representative of experiments performed in triplicate.

To investigate the sensitivity of the assay, the limit of detection (LOD) and limit of quantification (LOQ) were measured in multi-well plates for both systems (plain buffer and with DMSO, 15% *v*/*v*, Appendix B, Figure A2). The method turned out to be remarkable in detecting DFF in both media. The LOD and LOQ values for the DMSO–water system (8.4 μM and 25.5 μM, respectively) were slightly lower than the ones calculated in the plain buffer (Table 1).

### 2.3. Robustness of the Assay: Interference of Side Products

**Figure 6:**
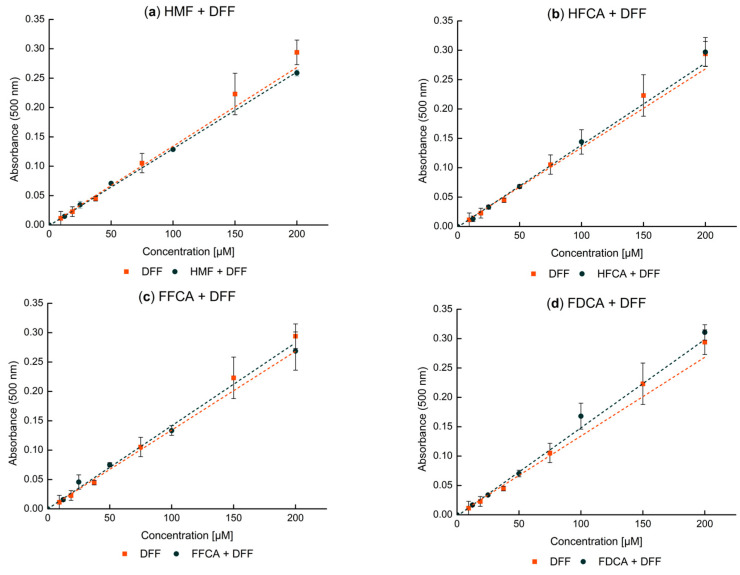
Effects of (**a**) HMF, (**b**) HFCA, (**c**) FFCA, and (**d**) FDCA on the colorimetric assay. Grey line: side product + DFF + PPD (as equimolar mixtures). Orange line: DFF + PPD. The data shown are representative of experiments performed in triplicate.

**Figure A3:**
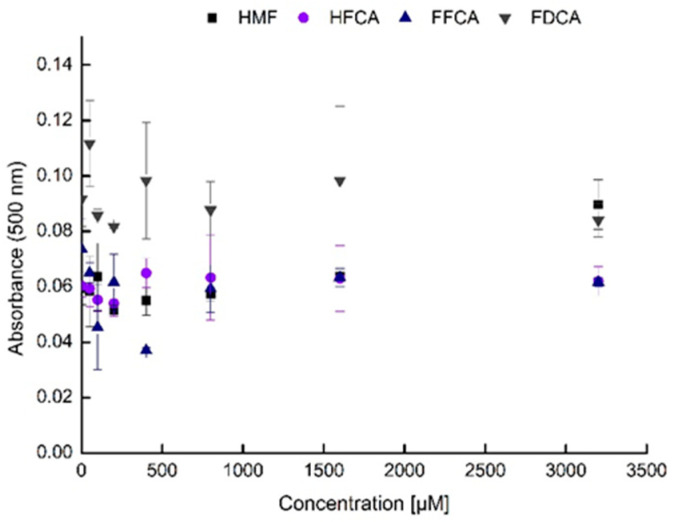
Interference of by-products (HFCA, FFCA, and FDCA) and HMF as residual substrate, measured at 500 nm under assay conditions in the presence of PPD (5 mM). The error is the result of three independent reaction assets.

The oxidative conversion of HMF towards DFF can result in a number of derivatives due to the presence of two reactive moieties, alcohol and aldehyde, which can form other compounds such 5-formyl-2-furancarboxylic acid (FFCA), 2,5-furandicarboxylic acid (FDCA), and 5-hydroxymethyl-2-furancarboxylic acid (HFCA), that could, therefore, be present in the reaction mixture (Figure 1) [86,87,88,89]. Hence, the interference of these side products, along with HMF as an unreacted substrate, with respect to DFF detection, was tested. Also, in this case, DMSO (15% *v*/*v*) was used to ensure the solubilization of all furan derivatives. For this purpose, different putative side product/DFF mixtures at various concentrations were prepared, simulating possible results in the enzymatic transformations. Each side product did not show any significant contribution to the absorbance at 500 nm when incubated with PPD (Appendix C, Figure A3). When combined with DFF, all of the investigated compounds did not interfere with the analyte detection (grey lines in panels, Figure 6a–d). As a reference, the response of the assay in the presence of only DFF was also reported (orange lines in panels, Figure 6a–d).

To further prove the robustness of the assay, the Z’ values were measured for each DFF–side product couple (see Experimental Section) [90,91,92]. This parameter offers a helpful indication of the effectiveness of the method, and it is an intrinsic factor that is used to assess the quality of an assay without the use of real samples, and thus, it can be further used for better development and optimization. The calculated values for each side product–DFF pair produced were in the range of 0.60–0.72, demonstrating the reliability of the method in identifying a positive hit (Table 2). Acceptable values are those >0.5, with our results appearing in the range of, e.g., those that Straathof and co-workers reported in the set-up of a high-throughput assay for amino acid decarboxylase activity detection [93].

This evidence shows the potential of our colorimetric assay as an easy, fast, and sensitive tool to be applied in the high-throughput screening of enzymes for the conversion of HMF to DFF in an aqueous solution under mild conditions. The analyte can be selectively detected at low concentrations, in the presence of DMSO as cosolvent, and without interferences of other possible co-products derived from HMF oxidation. However, a validation of the assay in the screening of enzyme libraries is still pending.

### 2.4. Application of the Colorimetric Assay: Kinetics Measurements

**Scheme 2:**
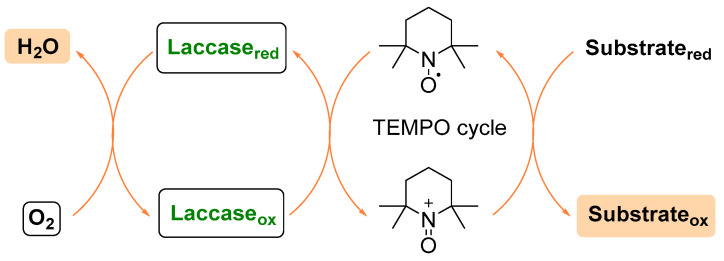
Overview of the laccase/TEMPO system to oxidize non-activated substrates.

After testing the assay on DFF and its mixture with different oxidized derivatives of HMF, the method was effectively applied on a real transformation. HMF oxidation into DFF was investigated by applying different LMSs. Among the described ones, the biocatalytic system with (2,2,6,6-tetramethylpiperidin-1-yl)oxyl (TEMPO) as a free radical mediator is one of the most applied [56,94,95,96], enabling the oxidation of non-activated substrates, such as alcohols and amines, and providing water as the only co-product (Scheme 2) [94,97,98].

**Figure 7:**
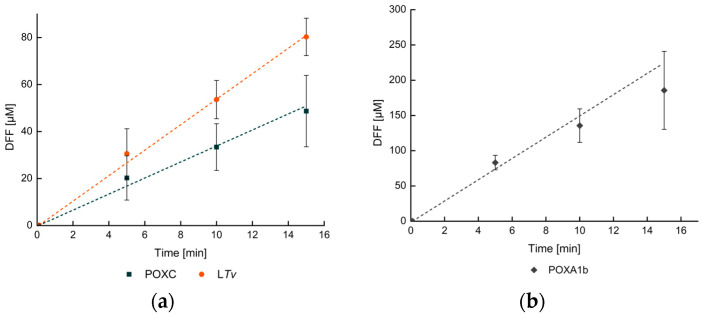
Time course of DFF production from HMF (40 mM) using the colorimetric assay at 30 °C under optimum conditions for each laccase: (**a**) POXC (pH 6.5, 0.25 U, grey dots) and L*Tv* (pH 5, 0.25 U, orange dots); (**b**) POXA1b (pH 5.5, 2.5 U), DMSO 15% *v*/*v*, and TEMPO (20 mol%) as mediator. Mol% is defined as moles of mediator per mol of substrate (%). The data shown are representative of experiments performed in triplicate and are presented as mean standard deviation.

Based on our previous results in the oxidation of primary alcohols into the corresponding aldehydes [97,99], including the formation of furfural from furfuryl alcohol [86] using the laccase/TEMPO system, herein, HMF oxidation into DFF was studied by employing three fungal laccases, one from *Trametes versicolor* (L*Tv*) [100], and two isolated from *Pleurotus ostreatus* (POXA1b and POXC) [101]. In fact, TEMPO/laccase systems have already been investigated for HMF transformations [102], providing FFCA [103] or FDCA [104,105] as the main achieved compounds in aqueous systems depending on the laccase and reaction conditions. Nevertheless, in the first stages of HMF oxidation, DFF is produced as a reaction intermediate. Therefore, to assess the validity of our colorimetric assay, the initial rates of the three different LMSs for DFF production were measured (Figure 7).

In our experiments, the mediator is responsible for the chemical oxidation of HMF, being then re-oxidized by the laccase. Its amount was kept constant in all tests (20 mol%). Accordingly, the comparison between the three different LMSs with regard to the initial rate for DFF appearance showed the different behavior and efficiency of the three laccases for TEMPO recycling. Concomitantly, DFF can undergo overoxidation within the time when the recycling system is particularly rapid. For this reason, lower amounts of enzyme were added for the most rapid systems (0.25 U for POXC and L*Tv*). Since the reaction was slower for the POXA1b system, the enzyme amount was increased up to 2.5 U to detect significant amounts of DFF in the same time slots. After the desired time was reached (5, 10, or 15 min), the medium was basified to favor the imine formation, and PPD (40 mM) was added. After 5 min at rt, the absorbance at 500 nm was measured. The initial rates for the different LMSs to produce DFF from HMF were then normalized by unit of enzyme (Table 3). 

In the L*Tv*-mediated system, great DFF accumulation was observed at short reaction times. Similarly, in the POXC-mediated reactions, good DFF amounts were produced at brief time frames; meanwhile, in the case of the POXA1b-mediated system, the accumulation of DFF was lower, directly connected to the rapidity of the laccases in re-oxidizing the radical TEMPO at short reaction times. The assay could be successfully applied to aqueous reaction mixtures performed at different pHs and buffer types, suggesting the possibility of using the assay for high-throughput screening and to assess the kinetics parameters for other HMF oxidizing enzymes, by determining DFF accumulation in the reaction mixture within the time.

## 3. Materials and Methods

Materials were purchased from Sarstedt (Nümbrecht, Germany). All chemicals were of the highest purity grade from commercial sources. Commercially available HMF was purchased from Manchester Organics (Runcorn, Cheshire, UK). DFF, FFCA, HFCA, and FDCA were purchased from Sigma-Aldrich (St. Louis, MO, USA). L*Tv* laccase was obtained from Sigma-Aldrich. Recombinant POXA1b from *Pleurotus ostreatus* expressed in the yeast *Pichia pastoris* [106,107], and POXC laccase produced and purified from *Pleurotus ostreatus* [108,109], were provided by BioPox srl (Napoli, Italy). The absorbance spectra of the mixtures were registered at room temperature using a Jasco V-530 UV/Vis spectrophotometer (Jasco International Co., Ltd., Tokio, Japan).

### 3.1. Colorimetric Assay Conditions

A suitable volume (150 µL) of a solution containing DFF and the other components at appropriate concentrations, dissolved in KPi buffer at 100 mM and pH 7.5 (when present, DMSO was used at 15% *v*/*v*), was transferred into each well of a 96-well plate. 1,4-Phenylenediamine was then added as reactant to each well (50 µL, 5 mM), shifting towards an orange color, according to the DFF concentration of the sample, to obtain a reddish precipitate. The plates were kept at room temperature for 5 min, and the increase in the absorbance at 500 nm was measured. The limit of absorption was set up to the formation of the precipitate, which caused a heterogeneous solution.

### 3.2. Determination of LOD and LOQ Values

LOD and LOQ were defined by IUPAC as “the limiting value of the true signal (related to some non-zero analyte concentration) which is significantly different from the blank signal value” and “the smallest concentration which can be quantitatively analyzed with reasonable reliability by the given procedure”, respectively [110]. LOD and LOQ values were calculated as three- and ten-fold, respectively, of the ratio between the standard deviation value of the DFF calibration curve and the numerical value of the slope [111].

### 3.3. Determination of Z’ Values

The Z’ factor is an intrinsic parameter that gives information about the quality of the assay without using real samples. The Z’ value is calculated using positive (DFF in our case) and negative reference controls (side products). Z’ values for by-product/DFF pairs close to 1.0 show the robustness of the method. To evaluate the overall performance of the assay and its application in an HTS format, values obtained either at 200 μM of DFF or the corresponding side product were used as positive and negative reference controls. The Z’ values of each side product–DFF pair were calculated using the following equation [90]:

$$Z^{\prime }=1-\frac{3SD_{\mathrm{DFF}}+3SD_{\mathrm{side}\;\mathrm{product}}}{\left|mean_{\mathrm{DFF}}-mean_{\mathrm{side}\;\mathrm{product}}\right|}$$

### 3.4. Laccase Activity Assay

Laccase activity was measured following the standard ABTS assay. ABTS (ε_420_ = 36,000 M^−1^·cm^−1^) test was performed at room temperature in 100 mM citrate buffer at pH 3.0, and 2 mM was the final concentration of the substrate. A suitable amount of enzyme necessary to obtain an absorbance of 0.5–1 was added after approximately 1 min. The increasing radical cation (ABTS^+•^) was tracked at 420 nm. One unit of laccase activity was defined as the enzyme amount that was able to oxidize 1 μmol of the substrate per minute [112].

### 3.5. Reaction Progress Curves for the Laccase–Mediator Systems and Initial Rate Measurements

To prevent leakages when spreading small volumes, the reagents were combined beforehand and then placed in the multiplate. The reaction progress curve (Figure 7) was obtained, dispensing 125 μL of the reaction mixture in each well, consisting of 30 U of laccase per mmol of substrate for POXA1b, 4 U per mmol of substrate for L*Tv* and POXC laccases, 20 mol% of TEMPO as mediator (mol of mediator per mol of substrate), and 40 mM of HMF starting concentrations (the concentration was selected after trials at different HMF amounts). Citrate buffers 100 mM and pH 5 and 5.5 were used for L*Tv* and POXA1b laccases, respectively, and KPi buffer 100 mM and pH 6.5 were used for POXC. All of the reaction mixtures were incubated in a rotary shaker into a multi-well plate (96 well) at 30 °C, 250 rpm. The reaction was stopped via enzyme inactivation by adding an aqueous 10 M NaOH solution (25 μL), and then PPD (40 mM) was added. The mixture was incubated for 5 min at room temperature, and finally, the absorbance at 500 nm was measured.

## 4. Conclusions

The development of accurate and easy-to-use high-throughput screening methods is a critical step in identifying novel biocatalysts or improving them for a specific application. In this work, we exploited the knowledge of an established reaction, a Schiff base formation followed by polymerization, to design a new colorimetric assay to directly detect DFF in aqueous reaction mixtures.

DFF is one of the high-value chemical platform compounds that are commonly obtained from HMF, so finding a method for its selective detection with respect to other possible by-products coming from HMF is highly appealing. In this manner, this assay may be efficiently used for screening libraries of redox biocatalysts looking for new or improved variants, as well as for assessing the kinetics properties of oxidative enzymes for DFF production. Hence, by employing a simple and cheap diamine compound such as 1,4-phenylenediamine, we have proven a sensitive, selective, and robust colorimetric assay towards DFF. The Z’ values, indeed, confirmed the absence of interferences due to the presence of HMF and other related furan derivatives that can be obtained after the oxidation of easily accessible HMF.

As a proof of concept, the performances of three different laccases with TEMPO applied to the synthesis of DFF were compared, displaying its utility in avoiding the expensive, time-consuming, and technically demanding analyses via chromatographic methods. As a fact, among the tested laccase–mediator systems, the assay showed that L*Tv* was the most efficient in producing DFF at short reaction times, with POXA1b being the less efficient one. All of this evidence reinforces the significance of the assay supporting not merely the search, but even the characterization of novel oxidative biocatalysts for the selective HMF oxidation into DFF.

## Figures and Tables

**Table 1:** Calibration curve regression analysis for determination of LOD and LOQ of the colorimetric assay in plain buffer or with 15% (*v*/*v*) DMSO.

Reaction System	Linear Range (μM)	Calibration Curve ^a^	SD of Calibration Curve	LOD (μM)	LOQ (μM)
Plain Buffer ^b^	6–200	A = 0.0008 × C − 0.0003	2.182 × 10^−3^	8.8	26.7
DMSO 15% (*v*/*v*)	6–300	A = 0.0014 × C − 0.0038	3.556 × 10^−3^	8.4	25.5

^a^ Absorbance value; C: concentration. ^b^ KPi buffer 100 mM, pH 7.5.

**Table 2:** Z’ values calculated for each couple of compounds (DFF + side product).

Side Product Combined with DFF	Z’ Value
HMF	0.72
HFCA	0.68
FFCA	0.60
FDCA	0.70

**Table 3:** Initial rate estimation for DFF production from HMF, applying the three LMSs. DFF was detected with the colorimetric method under the reported conditions, and its concentration was obtained via interpolation in the calibration curve.

Laccase	Reaction Medium ^a^	Initial Rate for DFF Production from HMF (μmol DFF·min^−1^·U^−1^)
L*Tv*	Citrate buffer, 100 mM, pH 5	21.7
POXC	Phosphate buffer, 100 mM, pH 6.5	13.9
POXA1b	Citrate buffer, 100 mM, pH 5.5	5.3

^a^ [HMF] = 40 mM, TEMPO (20 mol%), DMSO (15% *v*/*v*).

## Data Availability

The data presented in this study are available upon request from the corresponding authors.
